# Association between Dyslipidemia and Chronic Rhinosinusitis in a Korean Population

**DOI:** 10.3390/diagnostics11010026

**Published:** 2020-12-25

**Authors:** Jee Hye Wee, Chanyang Min, Min Woo Park, Soo Hwan Byun, Hyo-Jeong Lee, Chang Myeon Song, Bumjung Park, Hyo Geun Choi

**Affiliations:** 1 Department of Otorhinolaryngology-Head & Neck Surgery, Hallym University Sacred Heart Hospital, College of Medicine, Hallym University, Anyang 14068, Korea; weejh07@gmail.com (J.H.W.); hyobravo@gmail.com (H.-J.L.); bumjung426@gmail.com (B.P.); 2 Hallym Data Science Laboratory, College of Medicine, Hallym University, Anyang 14068, Korea; joicemin@naver.com; 3 Graduate School of Public Health, Seoul National University, Seoul 08826, Korea; 4 Department of Otorhinolaryngology-Head & Neck Surgery, Kangdong Sacred Heart Hospital, Seoul 05355, Korea; subintern@naver.com; 5 Department of Oral & Maxillofacial Surgery, Dentistry, Hallym University Sacred Heart Hospital, College of Medicine, Hallym University, Anyang 14068, Korea; purheit@daum.net; 6 Department of Otorhinolaryngology-Head & Neck Surgery, College of Medicine, Hanyang University, Seoul 04763, Korea; cmsong@hanyang.ac.kr

**Keywords:** sinusitis, dyslipidemias, nasal polyps, inflammation, population surveillance

## Abstract

This study aims to assess the relationship between chronic rhinosinusitis (CRS) and dyslipidemia in a Korean population. The population aged 40 years or over was selected from the Korean National Health Insurance Service-National Health Screening Cohort. CRS was defined if patients were treated ≥2 times with ICD-10 code (J32) and underwent head and neck computed tomography. Patients with CRS were classified as having nasal polyps (J33) or not. Dyslipidemia was defined if participants with the ICD-10 code (E78) were treated ≥2 times from 2002 to 2015. A total of 6163 patients with CRS were matched with 24,652 controls (1:4 ratio) for sex, age, income, and residence. The adjusted odds ratios (aORs) of a previous dyslipidemia in patients with CRS were analyzed by conditional logistic regression analysis, adjusted for confounding factors. The prevalence of dyslipidemia was significantly higher in participants with CRS (26.1%) than in the controls (20.6%) (*p* < 0.001). There was a significant positive association between CRS with/without nasal polyps and dyslipidemia (aOR = 1.36, 95%CI = 1.26–1.47, *p* < 0.001). The association between CRS and dyslipidemia was stronger for CRS without nasal polyps (aOR = 1.42, 95% CI = 1.28–1.57, *p* < 0.001) than for CRS with nasal polyps (aOR = 1.31, 95% CI = 1.17–1.47, *p* < 0.001). All age and sex subgroups exhibited consistent results. A personal history of dyslipidemia was associated with risk of CRS regardless of total cholesterol and the use of statins.

## 1. Introduction

Chronic rhinosinusitis (CRS) is a common chronic inflammatory disease of the sinonasal mucosa. The diagnosis of CRS often is based on clinical findings, including the duration of nasal symptoms, the characteristics of nasal discharge, and the presence of other symptoms. The endoscopic and/or computed tomography (CT) findings can distinguish between CRS with nasal polyps (CRScNP) and CRS without nasal polyps (CRSsNP) [1]. CRScNP has been associated with a type 2 inflammatory profile, while CRSsNP has been associated with a type 1 inflammatory profile [2,3]. The prevalence of CRS has been reported to range from 1% to 19% worldwide [4]. It is well known that CRS patients are more likely to have comorbidities such as asthma, chronic obstructive pulmonary disease, and cardiovascular disease [5,6,7]. In addition, some epidemiologic studies have reported relationships between CRS and components of metabolic syndrome, including obesity [8], diabetes [9], and hypertension [10].

Dyslipidemia is a disorder of lipid metabolism and an integral part of metabolic syndrome [11]. The World Health Organization estimated in 2008 that the global prevalence of dyslipidemia was 38.9% [12]. According to the Dyslipidemia Fact Sheets 2020, dyslipidemia is diagnosed according to one or more of the following characteristics: hyper-low-density lipoprotein (LDL)-cholesterol (serum LDL ≥ 160 mg/dL or taking lipid-lowering drug), hypo-high-density lipoprotein (HDL)-cholesterol (serum HDL ≤ 40 mg/dL), or hypertriglyceridemia (serum triglyceride ≥ 200 mg/dL), and a prevalence of dyslipidemia was reported as 38.4% (men: 45.6% and women: 31.3%) in Korea [13].

Several studies have suggested that statins have immunomodulatory and anti-inflammatory properties in addition to lipid-lowering effects. Patients taking statins had a 0.79-fold lower prevalence of CRS than those not taking statins in a study using a nationally representative sample in the United States [14]. However, an Italian case report reported that stains led to the development of eosinophilic polypoid rhinosinusitis [15]. Therefore, in view of similar inflammatory and immunological responses, possible associations between dyslipidemia and CRS can be considered, and the use of statins may affect analyses of the relationship between these conditions.

The object of this study was to compare the previous history of dyslipidemia between patients with CRS and controls after adjusting for the use of statins using a national sample cohort from the Korean population.

## 2. Materials and Methods

### 2.1. Study Population

The study was approved by the institutional review board (Ethics Committee of Hallym University, No. 2019-10-023). The need to obtain written informed consent was waived. The data from the Korean National Health Insurance Service (NHIS)-Health Screening Cohort was used. All Koreans have to register in the NHIS and the Korean Health Insurance Review and Assessment system managed all medical treatments in Korea. This NHIS included health insurance claim codes, diagnostic codes using the International Classification of Disease-10 (ICD-10), death records, socioeconomic data and health check-up data for each participant over the period from 2002 to 2015 [16].

### 2.2. Dyslipidemia (Exposure)

Dyslipidemia was defined if participants with the ICD-10 code (E78) were treated more than two times [17] from 2002 to 2015 and if the first day of diagnosis of dyslipidemia was before the day of the initiation of CRS treatment (index date).

### 2.3. Chronic Rhinosinusitis (Outcome)

CRS was defined with ICD-10 codes (J32). The participants who underwent head and neck CT (Claim codes: HA401-HA416, HA441-HA443, HA451-HA453, HA461-HA463, or HA471-HA473) and were treated more than two times with J32 were selected. CRScNP was defined with ICD-10 codes (J33) and CRSsNP was defined the other participants, as described in our previous studies [18,19].

### 2.4. Participant Selection

**Figure 1:**
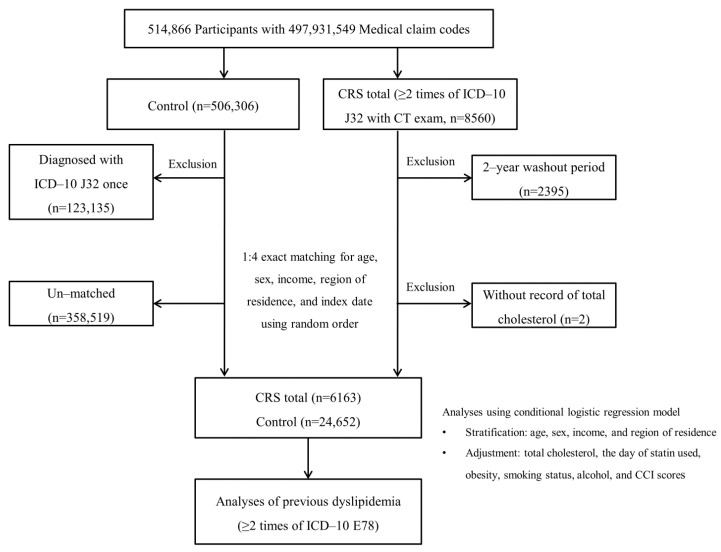
A flow diagram of the participant selection process. Among a total of 514,866 potential participants, 6163 chronic rhinosinusitis (CRS) participants were 1:4 matched with 24,652 controls for sex, age, income group, and residence. ICD-10 = International classification of disease-10; CT = Computed tomography; CCI = Charlson comorbidity index.

CRS patients (n = 8560) were selected from a total of 514,866 participants with 497,931,549 medical claim codes. Among the remaining participants (n = 506,306), those who had ever been treated with CRS of the ICD-10 code (J32) once from 2002 through 2015 (n = 123,135) were excluded. To select CRS patients who were diagnosed for the first time, we set a washout period of two years. CRS patients who were diagnosed from 2002 to 2003 (n = 2395) were excluded. Participants were excluded if they did not have records for total cholesterol (n = 2 for CRS patients). The control group was selected using a 1:4 matching with the CRS patients for sex, age, income group, and residence. To eliminate selection bias, the controls were selected in random order. The index date for each CRS patient was set as the time of treatment of CRS. The index date for controls was set as the index date of their matched CRS patient. Therefore, each matched CRS patient had a control with the same index date. During the matching process, 358,519 controls were eliminated. Finally, 6163 participants with CRS were 1:4 matched with 24,652 controls (Figure 1). Among the CRS patients, 2958 was those with CRScNP and 3205 was those with CRSsNP.

### 2.5. Covariates

Age was grouped into ten classes with five-year intervals: 40 to 44 years old, 45 to 49 years old…, over 85 years old. Income groups were classified as five classes (class 1 [lowest income]-5 [highest income]). The region of residence was grouped into urban (Seoul, Busan, Daegu, Incheon, Gwangju, Daejeon, and Ulsan) and rural (Gyeonggi, Gangwon, Chungcheongbuk, Chungcheongnam, Jeollabuk, Jeollanam, Gyeongsangbuk, Gyeongsangnam, and Jeju) areas. Obesity, as measured by the body mass index (BMI, kg/m^2^), was categorized as <18.5 (underweight), ≥18.5 to <23 (normal), ≥23 to <25 (overweight), ≥25 to <30 (obese I), and ≥30 (obese II) [20]. Missing BMI values (n = 12) were replaced by the mean BMI of the included subjects. Smoking status was categorized into nonsmoker, past smoker, and current smoker. Alcohol use was categorized on the basis of the frequency of alcohol consumption (<1 time a week and ≥1 time a week). The Charlson Comorbidity Index (CCI) is a commonly used indicator to determine the disease burden based on 17 comorbidities [21]. The days of statin use were calculated for the 730 days (2 years) prior to the index date. Serum total cholesterol (mg/dL) was measured.

### 2.6. Statistics

The general characteristics between the CRScNP/CRSsNP groups and controls were compared using the chi-square test for categorical variables and the independent *t* test for continuous variables.

To analyze the ORs with CIs for dyslipidemia in patients with CRScNP/CRSsNP compared to the controls, conditional logistic regression was applied, with the crude model, model 1 (adjusted for total cholesterol and the days of statin use), and model 2 (adjusted for total cholesterol, the days of statin use, obesity, alcohol drinking, smoking status, and CCI scores). The analysis was stratified by sex, age, income group, and residence.

Subjects were classified by age and sex (<60-year-old men and women; ≥60-year-old men and women) and subgroup analyses with the crude model, model 1, and model 2 were performed.

Additionally, multiple linear regression was performed to calculate the estimated value and 95% CIs for the days of statin use and total cholesterol in patients with CRScNP/CRSsNP compared to the controls (Supplementary Table S1).

Two-tailed analyses were carried out, and *p* values ≤0.05 were defined as significant. SAS 9.4 (SAS Institute Inc., Cary, NC, USA) was used.

## 3. Results

### 3.1. General Characteristics

Table 1 shows the general characteristics of the participants. Sex, age, income group, and residence were identical between the CRS and control groups due to matching (all *p* = 1.000). Compared with the controls, CRS patients were more likely to be obese (*p* = 0.003) and nonsmokers (*p* < 0.001) and had higher CCI scores (*p* < 0.001). The prevalence of dyslipidemia was significantly higher in the CRS patients (26.1%) than in the controls (20.6%) (*p* < 0.001). The CRS group had more days of statin use (57.8 days vs. 44.3 days, *p* < 0.001) and a lower total cholesterol level (197.1 mg/dL vs. 199.0 mg/dL, *p* = 0.001) than the controls.

### 3.2. Association between CRS and Dyslipidemia

Among all participants, there was a significant positive association between CRS and dyslipidemia (adjusted odds ratio [aOR] = 1.36, 95% confidence interval [CI] = 1.26–1.47, *p* < 0.001, Table 2). When analyzing the CRScNP and CRSsNP groups separately, the association between CRS and dyslipidemia was stronger in the CRSsNP group (aOR = 1.42, 95% CI = 1.28–1.57, *p* < 0.001) than in the CRScNP group (aOR = 1.31, 95% CI = 1.17–1.47, *p* < 0.001).

### 3.3. Subgroup Analyses According to the Age and Sex

**Figure 2:**
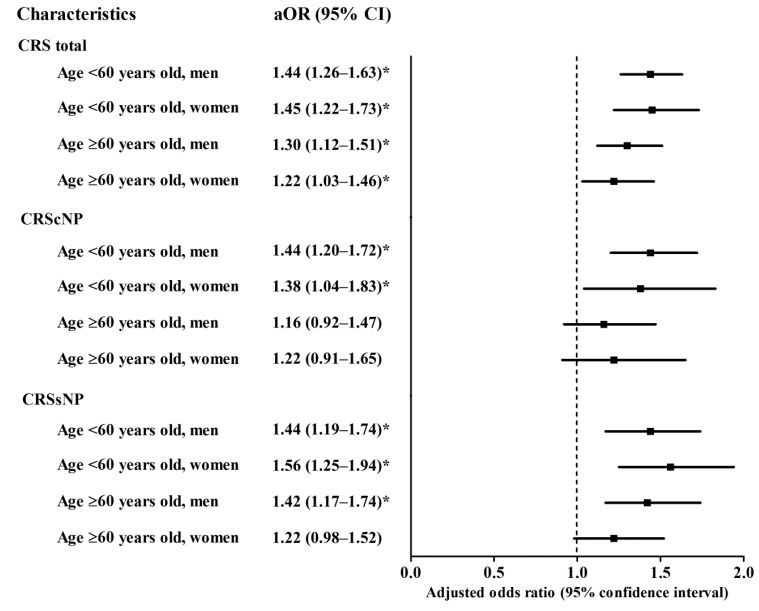
Subgroup analyses by the age and sex. The adjusted odds ratio (aOR) with 95% confidence interval for dyslipidemia in chronic rhinosinusitis (CRS) total/CRS with nasal polyps (CRScNP)/CRS without nasal polyps (CRSsNP) groups compared to each control group. * Significance at *p* < 0.05.

In the subgroup analyses stratified by age and sex, all aORs for dyslipidemia were significantly higher in the CRS group than in the controls (Figure 2, Supplementary Table S2). The aORs were 1.44 (95% CI = 1.26–1.63, *p* < 0.001) in <60-year-old men, 1.45 (95% CI = 1.22–1.73, *p* < 0.001) in <60-year-old women, 1.30 (95% CI = 1.12–1.52, *p* = 0.001) in ≥60-year-old men, and 1.22 (95% CI = 1.03–1.46, *p* = 0.025) in ≥60-year-old women.

## 4. Discussion

This study showed that CRS is associated with dyslipidemia. Few studies have reported such an association. A Taiwanese population-based study showed that subjects with CRS had increased rates of various comorbidities, and among them, the aOR for hyperlipidemia was 1.34 (95% CI = 1.24–1.43) in subjects with CRS compared to those without CRS [22]. This result is consistent with our finding, but the authors conducted conditional logistic regression analyses based on age and sex groups, adjusting only for sociodemographic characteristics (region, income, and level of urbanization). A study using data from the 5th Korean National Health and Nutrition Examination Survey reported that the prevalence of CRS was higher in patients with metabolic syndrome (a reduced high-density lipoprotein level, a high triglyceride level, and elevated blood pressure, 14.15%) than in those without metabolic syndrome (10.16%) [23]. However, they did not consider the use of statins. In the present study, we adjusted for several potential confounders, including the days of statin use, levels of total cholesterol, and other comorbidities.

We hypothesize that there are several possible mechanisms underlying the association between CRS and dyslipidemia. First, both CRS and dyslipidemia are risk factors for cardiovascular disease. A meta-analysis reported that CRS is associated with a higher risk of stroke (pooled relative risk = 1.79, 95% CI = 1.34–2.40) [24], and a Taiwanese population-based study showed that patients with CRS were at higher risk for acute myocardial infarction (hazard ratio = 1.70, 95% CI = 1.52–1.91) in six years of follow-up. In a large international case-control study, dyslipidemia was recognized as a prominent risk factor for cardiovascular disease (OR = 3.25, population attributable risk = 49.2%) [25]. Second, both CRS and dyslipidemia have underlying associations with endothelial dysfunction [26,27]. Previous studies have shown that chronic inflammation has negative effects on the function and integrity of endothelial cells, resulting in accelerated atherogenesis [28,29]. Oxidative stress has been reported to be an important factor determining coronary endothelial dysfunction in patients with hypercholesterolemia and atherosclerosis [30]. In addition, CRS has been found to be associated with oxidative stress and low glutathione (antioxidant) levels [31].

The etiology of CRS is multifactorial, and several hypotheses have been proposed involving a number of host or environmental factors [32]. Among them, the ‘immune barrier hypothesis’ assumes that chronic inflammation is associated with defects in the mechanical barrier and innate immune response. Defects in the mechanical barrier and the innate immune response can render an individual more vulnerable to CRS caused by common microbes. Hypercholesterolemia is known to increase the levels of proinflammatory cytokines, cellular adhesion molecules, and inflammation-sensitive plasma proteins and is associated with the enhanced expression of proinflammatory mechanisms [33,34,35]. Such proinflammatory host factors may be linked to dyslipidemia in CRS patients. In addition, a recent review suggested that obesity is a factor that increases the incidence of CRS [36]. A previous study using data from the National Health and Nutritional Examination Survey 1999–2002 showed that dyslipidemia is the most common comorbidity associated with various BMI categories and that the prevalence of dyslipidemia increases with increasing body weight [37]. In a study from the China National Stroke Screening and Prevention Project, obesity was associated with a high risk of all components of dyslipidemia [38].

In the present study, the association between dyslipidemia and CRS was significant in both the CRSsNP (Th1 dominant) and CRScNP (Th2 dominant) groups. It can be explained by the results of an Indian study using the Chennai Urban Rural Epidemiology Study that showed an increased in both Th1 and Th2 cytokines in subjects with metabolic syndrome [39]. Furthermore, the association between dyslipidemia and CRS was higher in the CRSsNP group (aOR = 1.42) than in the CRScNP group (aOR = 1.31). We could not exactly explain the mechanism underlying this association, because few studies have addressed the Th immune response in dyslipidemia, and conflicting data have been reported regarding the association between metabolic syndrome and immunological dysregulation [40,41,42]. However, previous studies have shown that there is an initial dominance of the Th1 pattern during the progression of the disease and switching to Th2 pattern with increasing levels of cholesterol [41,42]. Further studies are needed to better understand the immunologic mechanism.

This study has some limitations. First, we had no data on the severity of CRS, including the sinonasal symptoms or Lund-Mackey scores. Second, various medications used to treat CRS, such as antihistamines, antibiotics, or intranasal corticosteroids, could contribute to the development of inflammatory diseases. Third, we only adjusted for serum level of total cholesterol. However, a recent prospective cohort study reported that unadjusted baseline serum total cholesterol, LDL cholesterol, and triglyceride levels were positively associated with the risk of total cardiovascular events, but after adjusting for confounding factors, a significant direct association remained only between total cholesterol and the risk of myocardial infarction [43]. Despite these limitations, the strength of this study is that we used a large representative nationwide cohort dataset with diagnoses based on ICD-10 codes, making the analysis less susceptible to recall bias than most epidemiologic studies using self-reported diagnoses.

## 5. Conclusions

A personal history of dyslipidemia was significantly associated with risk of CRS, regardless of total cholesterol and the use of statins.

## Figures and Tables

**Table 1:** General Characteristics of Participants.

Characteristics	Total Participants		
	CRS total	Control	*p*-Value
Age (years old), n (%)			1.000
40–44	232 (3.8)	928 (3.8)	
45–49	972 (15.8)	3888 (15.8)	
50–54	1339 (21.7)	5356 (21.7)	
55–59	1303 (21.1)	5212 (21.1)	
60–64	986 (16.0)	3944 (16.0)	
65–69	712 (11.6)	2848 (11.6)	
70–74	388 (6.3)	1552 (6.3)	
75–79	170 (2.8)	680 (2.8)	
80–84	50 (0.8)	200 (0.8)	
85+	11 (0.2)	44 (0.2)	
Sex, n (%)			1.000
Male	3786 (61.4)	15,144 (61.4)	
Female	2377 (38.6)	9508 (38.6)	
Income group, n (%)			1.000
1 (lowest)	756 (12.3)	3024 (12.3)	
2	727 (11.8)	2908 (11.8)	
3	932 (15.1)	3728 (15.1)	
4	1349 (21.9)	5396 (21.9)	
5 (highest)	2399 (38.9)	9596 (38.9)	
Residence, n (%)			1.000
Urban	2856 (46.3)	11,424 (46.3)	
Rural	3307 (53.7)	13,228 (53.7)	
Obesity, n (%)			0.003 ^1^
Underweight	104 (1.7)	508 (2.1)	
Normal	2003 (32.5)	8456 (34.3)	
Overweight	1829 (29.7)	6815 (27.6)	
Obese I	2052 (33.3)	8144 (33.0)	
Obese II	175 (2.8)	729 (3.0)	
Smoking status, n (%)			<0.001 ^1^
Nonsmoker	4047 (65.7)	15,960 (64.7)	
Past smoker	908 (14.7)	3226 (13.1)	
Current smoker	1208 (19.6)	5466 (22.2)	
Alcohol drinking, n (%)			0.198
<1 time a week	4034 (65.5)	15,920 (64.6)	
≥1 time a week	2129 (34.5)	8732 (35.4)	
CCI score, n (%)			<0.001 ^1^
0	3983 (64.6)	17,809 (72.2)	
1	1008 (16.4)	3124 (12.7)	
2	557 (9.0)	1722 (7.0)	
3	273 (4.4)	808 (3.3)	
≥4	342 (5.6)	1189 (4.8)	
Dyslipidemia, n (%)	1608 (26.1)	5067 (20.6)	<0.001 ^1^
Days of statin use (day), mean (SD)	57.8 (168.3)	44.3 (148.5)	<0.001 ^2^
Total cholesterol (mg/dL) mean (SD)	197.1 (37.2)	199.0 (37.9)	0.001 ^2^

CCI = Charlson comorbidity index; CRS = Chronic rhinosinusitis; SD = standard deviation. ^1^ Chi-square test. Significance at *p* < 0.05. ^2^ Independent *t* test. Significance at *p* < 0.05.

**Table 2:** Crude and adjusted odds ratios (95% confidence interval) for dyslipidemia in CRS total/CRScNP/CRSsNP groups compared to each control group.

Characteristics	Odds Ratios for Dyslipidemia					
	Crude ^2^	*p*-Value	Model 1 ^2,3^	*p* -Value	Model 2 ^2,4^	*p* -Value
CRS total (n = 6163)	1.39 (1.30–1.48)	<0.001 ^1^	1.37 (1.27–1.48)	<0.001 ^1^	1.36 (1.26–1.47)	<0.001 ^1^
Control (n = 24,652)	1.00		1.00		1.00	
CRScNP (n = 2958)	1.28 (1.15–1.41)	<0.001 ^1^	1.33 (1.18–1.49)	<0.001 ^1^	1.31 (1.17–1.47)	<0.001 ^1^
Control (n = 11,832)	1.00		1.00		1.00	
CRSsNP (n = 3205)	1.48 (1.36–1.62)	<0.001 ^1^	1.42 (1.28–1.57)	<0.001 ^1^	1.42 (1.28–1.57)	<0.001 ^1^
Control (n = 12,820)	1.00		1.00		1.00	

CCI = Charlson comorbidity index; CRS = chronic rhinosinusitis; CRScNP = CRS with nasal polyps; CRSsNP = CRS without nasal polyps. ^1^ Conditional logistic regression model, Significance at *p* < 0.05. ^2^ Models stratified by sex, age, income group, and residence. ^3^ Model 1 was adjusted for total cholesterol and days of statin use. ^4^ Model 2 was adjusted for total cholesterol, days of statin use, obesity, smoking status, alcohol drinking, and CCI scores.

## Data Availability

The data presented in this study are available from the Korea National Health Insurance Sharing Service (https://nhiss.nhis.or.kr) subject to their requirements and fees.

## Supplementary Materials

The following are available online at https://www.mdpi.com/2075-4418/11/1/26/s1, Table S1: Multiple linear regression model (estimated value [95% confidence interval]) for the days of statin use and total cholesterol in the CRS total/CRScNP/CRSsNP groups compared to the control group, Table S2: Subgroup analyses of crude and adjusted odds ratios (95% confidence interval) for dyslipidemia in the CRS total/CRScNP/CRSsNP groups compared to each control group according to the age and sex.
