# Supplementary material for: Association between Dyslipidemia and Chronic Rhinosinusitis in a Korean Population
Source: Diagnostics (Basel). 2020 Dec 25;11(1):26. doi: 10.3390/diagnostics11010026 (PMC7823289; doi:10.3390/diagnostics11010026)
Supplement: Supplementary file 1 [file diagnostics-11-00026-s001.pdf]

Table S1. Multiple linear regression model (estimated value [95% confidence interval]) for the days of statin use and total cholesterol in the CRS total/CRScNP/CRSsNP groups compared to the control group.

**Characteristics**	**Multiple linear regression**[2,3]	**P-value**
CRS total		
Days of statin use	2.440 (–1.238 to 6.118)	0.194
Total cholesterol	–2.199 (–3.224 to –1.173)	<0.001[1]
CRScNP		
Days of statin use	–2.507 (–7.279 to 2.266)	0.303
Total cholesterol	–1.606 (–3.092 to –0.120)	0.034[1]
CRSsNP		
Days of statin use	6.735 (1.215 to 12.255)	0.017[1]
Total cholesterol	–2.716 (–4.133 to –1.299)	<0.001[1]

CCI=Charlson comorbidity index; CRS=Chronic rhinosinusitis; CRScNP=CRS with nasal polyps; CRSsNP=CRS without nasal polyps

[1] Linear regression model, Significance at $P < 0.05$

[2] A model stratified by sex, age, income group, and residence.

[3] A model adjusted for dyslipidemia history, obesity, smoking status, alcohol drinking, and CCI scores. Total cholesterol was additionally adjusted if days of statin use was dependent variable. Days of statin use was additionally adjusted if total cholesterol was dependent variable.

Table S2. Subgroup analyses of crude and adjusted odds ratios (95% confidence interval) for dyslipidemia in the CRS total/CRScNP/CRSsNP groups compared to each control group according to the age and sex.

Characteristics	Odds ratios for dyslipidemia					
	Crude†	P-value	Model 1[2,3]	P-value	Model 2[2,4]	P-value
Age <60 years old, men						
CRStotal vs. control (n=12,260)	1.44 (1.28–1.62)	<0.001[1]	1.45 (1.28–1.65)	<0.001[1]	1.44 (1.26–1.63)	<0.001[1]
CRScNP vs. control (n=6930)	1.40 (1.19–1.64)	<0.001[1]	1.46 (1.22–1.74)	<0.001[1]	1.44 (1.20–1.72)	<0.001[1]
CRSsNP vs. control (n=5330)	1.49 (1.26–1.76)	<0.001[1]	1.45 (1.20–1.75)	<0.001[1]	1.44 (1.19–1.74)	<0.001[1]
Age <60 years old, women						
CRS total vs. control (n=6970)	1.37 (1.18–1.60)	<0.001[1]	1.46 (1.22–1.73)	<0.001[1]	1.45 (1.22–1.73)	<0.001[1]
CRScNP vs. control (n=2990)	1.10 (0.86–1.40)	0.436	1.36 (1.03–1.79)	0.033[1]	1.38 (1.04–1.83)	0.025[1]
CRSsNP vs. control (n=3980)	1.59 (1.31–1.92)	<0.001[1]	1.58 (1.26–1.96)	<0.001[1]	1.56 (1.25–1.94)	<0.001[1]
Age ≥60 years old, men						
CRS total vs. control (n=6670)	1.38 (1.21–1.57)	<0.001[1]	1.32 (1.13–1.53)	<0.001[1]	1.30 (1.12–1.51)	0.001[1]
CRScNP vs. control (n=3035)	1.21 (0.99–1.47)	0.064	1.21 (0.96–1.52)	0.113	1.16 (0.92–1.47)	0.210
CRSsNP vs. control (n=3635)	1.53 (1.29–1.83)	<0.001[1]	1.41 (1.16–1.72)	0.001[1]	1.42 (1.17–1.74)	0.001[1]
Age ≥60 years old, women						
CRS total vs. control (n=4915)	1.32 (1.15–1.53)	<0.001[1]	1.24 (1.04–1.47)	0.018[1]	1.22 (1.03–1.46)	0.025[1]
CRScNP vs. control (n=1835)	1.30 (1.01–1.66)	0.039[1]	1.22 (0.90–1.63)	0.198	1.22 (0.91–1.65)	0.183
CRSsNP vs. control (n=3080)	1.34 (1.12–1.61)	0.001[1]	1.25 (1.01–1.55)	0.043[1]	1.22 (0.98–1.52)	0.071

CCI=Charlson comorbidity index; CRS=Chronic rhinosinusitis; CRScNP=CRS with nasal polyps; CRSsNP=CRS without nasal polyps

[1] Conditional logistic regression model, Significance at P<0.05

[2] Models stratified by sex, age, income group, and residence.

[3] Model 1 was adjusted for total cholesterol and days of statin use.

[4] Model 2 was adjusted for total cholesterol, days of statin use, obesity, smoking status, alcohol drinking, and CCI scores.
